# Clinical pharmacokinetics and dose recommendations for posaconazole gastroresistant tablets in children with cystic fibrosis

**DOI:** 10.1093/jac/dkab312

**Published:** 2021-08-30

**Authors:** Siân Bentley, Jane C Davies, Silke Gastine, Jackie Donovan, Joseph F Standing

**Affiliations:** 1 Pharmacy Department, Royal Brompton Hospital, London, UK; 2 National Heart and Lung Institute, Imperial College London, London, UK; 3 Paediatric Respiratory Medicine Department, Royal Brompton Hospital, London, UK; 4 Infection, Immunity and Inflammation, Great Ormond Street Institute of Child Health, University College London, London, UK; 5 Clinical Biochemistry Department, Royal Brompton Hospital, London, UK; 6 Pharmacy Department, Great Ormond Street Hospital for Children, NHS Foundation Trust, London, UK

## Abstract

**Objectives:**

To investigate the population pharmacokinetics of posaconazole gastroresistant tablets in children with cystic fibrosis (CF) and perform simulations to recommend optimal doses.

**Patients and methods:**

Children from a paediatric CF centre who had received posaconazole tablets and underwent therapeutic drug monitoring were identified from pharmacy records. Relevant clinical data were collated from case notes and electronic patient records and used to develop an allometrically scaled population pharmacokinetic model. A stepwise covariate model-building exercise evaluated the influence of interacting medicines and liver function.

**Results:**

One hundred posaconazole serum concentrations were collected from 37 children with a median age of 14 years (range 7–17). Posaconazole pharmacokinetics were adequately described by a one-compartment model with inter-individual variability on clearance. Dose simulations demonstrated a 77%–83% probability of attaining a trough target of 1 mg/L with a dose of 300 mg every 12 h for two doses then 300 mg once daily (OD) in children aged 6–11 years; and 86%–88% with a dose of 400 mg every 12 h for two doses then 400 mg OD in adolescents aged 12–17 years. This dose scheme also yielded a 90% probability of achieving an AUC of 30 mg·h/L. AUC and trough concentration were highly correlated (r^2^ = 0.98). Simulations showed that trough concentrations of >0.75 mg/L would exceed an AUC of 30 mg·h/L in 90% of patients.

**Conclusions:**

A starting dose of 300 mg OD in those aged 6–11 years and 400 mg OD in those aged 12–17 years (following loading doses) yields a 90% probability of attaining an AUC of 30 mg·h/L.

## Introduction

Cystic fibrosis (CF) is a common autosomal recessive genetic disorder, resulting from the defective CF transmembrane conductance regulator (CFTR) gene, which causes abnormal mucous secretions in multiple organs, notably the lungs, liver and pancreas. Lung disease is characterized by chronic pulmonary infection by opportunistic bacteria and fungi, leading to inflammation and a progressive decline in lung function. *Aspergillus fumigatus* is one of the most commonly isolated fungi in sputum from CF patients.^1^ Reported prevalence in children varies between 16% and 42%.^2^^,^^3^ In 2019, UK CF Registry data reported *Aspergillus* grown in 9.4% of children (aged 0–15 years) in the 12 months prior to the 2019 annual review. This is proportionally higher in older age groups, with positive sputum samples found in 10.1% of children aged 8–11 years and 18.9% in those aged 12–15 years.^4^ Other fungi commonly isolated in CF include *Scedosporium* species and *Exophiala dermatitidis*.^1^

Triazole antifungal medicines, such as posaconazole, are used for the treatment of lung disease caused by these fungi in children with CF. Although there are no national or international guidelines specific to CF, this is in line with international guidelines for the wider population.^5–7^ Posaconazole is not licensed in children <18 years old in the UK and EU, or those <13 years old in the USA; however, it is increasingly being used in children with CF due to the poor tolerability, toxicity and high inter-individual variability (IIV) in drug levels of the alternative azoles, itraconazole and voriconazole.^8^^,^^9^ Due to the lack of data supporting posaconazole dosing regimens in children, as well as the aforementioned IIV exhibited in other azoles, therapeutic drug monitoring (TDM) is routinely carried out. The ESCMID-ECMM-ERS guidelines for the diagnosis and management of *Aspergillus* diseases and British Society of Medical Mycology antifungal TDM guidelines suggest a posaconazole treatment target trough concentration of >1 mg/L, with no upper limit yet shown to be associated with toxicity.^7^^,^^10^

People with CF are considered to be a unique population, displaying altered pharmacokinetics for some drugs, most likely as a result of comorbidities affecting drug absorption and disposition.^11^ This has been borne out in several studies; in a small prospective pharmacokinetic study of itraconazole in paediatric CF patients, many failed to achieve adequate serum concentrations with standard doses.^12^ More recently, in a retrospective observational study evaluating posaconazole plasma concentrations in lung transplant recipients, *C*_min_ for the tablet formulation was found to be 48% lower in patients with CF.^13^ Together with a paucity of paediatric pharmacokinetic data for medicines new to the market on which to base dosing regimens, the use of inappropriate dosing regimens may reduce the effectiveness of posaconazole in this cohort of patients, increase the risk of adverse effects and potentially drive resistance; in a tertiary institution, 16.2% of *A. fumigatus* isolates from CF patients were found to be resistant to azole antifungals.^14^

A recently published population pharmacokinetic model provided dose recommendations for a liquid formulation of posaconazole in immunocompromised children.^15^ Given the potential differences in the pharmacokinetics of some medicines in people with CF, and extensive use of the tablet formulation of posaconazole in this population, with known pharmacokinetic differences to the liquid formulation,^16^ our study aimed to develop a population pharmacokinetic model of posaconazole gastroresistant tablets in a cohort of paediatric CF patients. This was used to evaluate the influence of patient characteristics, including interacting medicines, on posaconazole exposure. We then performed simulations to produce dosing recommendations in this unique population.

## Patients and methods

### Study design

Retrospective patient data collected during the routine care of children aged between 5 and 17 years (both inpatients and outpatients) within the paediatric CF clinic at a tertiary centre, who had received posaconazole gastroresistant tablets for treatment of CF-related fungal lung disease between 2014 and 2020, were collated. The study and use of anonymized clinical data was approved by the Trust’s Research and Development Office and Health Research Authority, reference number 20/HRA/3760. As the study involved the retrospective use of anonymized data, formal participation/parental consent was not required. Patients were identified by matching pharmacy and clinical biochemistry records by pharmacy staff who routinely care for children with CF. Patients who had at least one posaconazole TDM sample taken were included. The time and date of the posaconazole TDM sample, along with the reported concentration, were extracted from electronic patient records. Samples that were below the limit of quantification in the analysis were recorded in the dataset as LOQ/2. For inpatients, dosing history was taken from the electronic nursing administration record, capturing doses given in the preceding 48 h before sampling. For outpatients, the time of the last dose, if not recorded, was presumed to be at 8 am on the day of the sample and assumed to be at steady state based on the patient’s drug history. The model was run with and without these samples to check if this assumption led to biased parameter estimates. Additional data collected from electronic patient records were basic demographic information, liver function at the time of sampling, concomitant interacting medications, use of concomitant proton pump inhibitors and histamine H_2_-receptor antagonists, the indication for posaconazole, details of the dosing regimen (including formulation taken) and sampling time/date. Samples were excluded from the analysis if sample timing was not known or if preceding doses were identified as not administered.

### Analytical method

Posaconazole analysis was carried out using 2D TurboFlow™ HPLC tandem MS (2D HPLC-MS/MS). This was on a Thermo Fisher Scientific Transcend™ prior to 2017 and a Prelude SPLC™ HPLC system coupled with a TSQ Endura™ triple quadruple mass spectrometer (Thermo Fisher Scientific, CA, USA) thereafter.

Analysis was carried out by Trust laboratories using in-house methodology, developed in line with FDA and EMA guidelines.^17^^,^^18^ The intra- and inter-assay precision across the reporting range of 0.2 to 8 mg/L were below 5.2%.

Samples were stored at −20°C prior to analysis. Posaconazole was extracted from serum via protein precipitation with acetonitrile. Posaconazole-d4 was used as the corresponding internal standard. Chromatography mobile phases were 10 nM ammonium acetate in ultrapure water (UP-H_2_O) and 10 nM ammonium acetate in methanol and posaconazole was detected using atmospheric chemical ionization in positive ion mode.

### Population pharmacokinetic model building

Parameters for a one-compartment model assuming first-order absorption and elimination were estimated using non-linear mixed-effects modelling with NONMEM (Version 7.4; ICON Development Solutions, Ellicott City, MD, USA),^19^ using the FOCE with interaction algorithm. IIV was tested for clearance assuming a log-normal distribution. The residual error included additive and proportional terms.

Allometric size scaling of clearance and volume terms were added *a priori*^13^ in the following form:

$$p_{i}=\theta_{p}{\left(\frac{c_{i}}{\bar{c}}\right)}^{\theta_{c}}$$

where $p_{i}$ is the individual parameter of interest, $c_{i}$ is the individual value of the covariate and $\bar{c}$ is the typical value of the covariate in the population. In the fixed allometric weight scaling, $c_{i}$ was the individual body weight, $\bar{c}$ was set to 70 kg and $\theta_{c}$ was 0.75 for clearance and 1 for central volume.

For nested models, significant model improvement through additional parameters was evaluated with the likelihood ratio test, the difference in −2 log-likelihood (objective function value, OFV, in NONMEM) of the models being asymptotically $\chi_{1}^{2}$ distributed. Covariates were added if the likelihood ratio test indicated a significant improvement in fit at the level of *P* < 0.01. A stepwise covariate model (SCM)-building exercise with Perl Speaks NONMEM (PsN)^20^ considered forward inclusion with a *P* value of ≤0.05 and backwards elimination with a *P* value of ≤0.01. The following covariates were tested on clearance: liver function abnormality (if any of ALT, alkaline phosphatase, AST, GGT or bilirubin were outside of the normal reference ranges); and potential interacting medicines that were identified in patients included in the dataset: Orkambi^®^ (lumacaftor/ivacaftor), rifampicin, rifabutin, clarithromycin, histamine H_2_-receptor antagonists and proton pump inhibitors.

### Dose simulations

Dosing simulations were performed in R.^21^ The multivariate mean, variance and covariance between age and weight were estimated from the patient demographics and then simulations performed to generate 1000 age–weight pairs for each simulation scenario. Using both this dataset and the final model, simulations were generated to assess the probability of attaining a trough target concentration of 1 mg/L^7^^,^^10^ and an alternative target of an AUC of 30 mg·h/L.^22^^,^^23^

### Model evaluation

Final model evaluation consisted of plotting predictions versus observations, and conditional weighted residuals versus time and predictions. A visual predictive check with 1000 samples was performed to check predictive performance, and a non-parametric bootstrap (1000 samples) was conducted to evaluate parameter robustness.

## Results

### Study population

The initial dataset included 109 posaconazole serum concentrations from 41 children, obtained during 65 treatment courses. From this, nine serum concentrations were excluded (three where we were unable to ascertain the timing of the sample; three due to incomplete dose history, including uncertainty on whether doses had been administered; two below the limit of quantification in a patient known to have been non-adherent; and one where it was identified that the patient had missed a number of the preceding doses). The final dataset therefore included 100 levels from 37 children, with a median age of 14 years (range 7–17). There were 29 samples taken from children aged 7–11 years, with median weight of 31.5 kg (range 25–58). The median posaconazole serum concentration for this age group was 3.08 mg/L (range 0.2–8.91) from a median dose of 300 mg (range 100–300). Of 71 samples taken from children aged 12–17 years, with median weight of 50 kg (range 34.7–82.8), the median posaconazole serum concentration was 2.07 mg/L (range 0.2–4.96) from a median dose of 300 mg (range 200–600). There were 47 samples from outpatients where the time of the last dose was presumed to be at 8 am on the day of the sample. Study population characteristics are detailed in Table 1.

**Table 1.** Demographics of patients included in pharmacokinetic analysis

Variable	Included patients (*n* *=* 37)
TDM samples, *n*	100
Samples/patient, *n*, median (range)	2 (1–9)
Organism/condition being treated at the time of the sample: ABPA/*Aspergillus*/*Scedosporium*/*Exophiala*	53/17/26/4
Age, years (range)	14 (7–17)
Weight, kg (range)	45.55 (25–82.8)
Age 6–11 years	31.5 (25–58)
Age 12–17 years	50 (34.7–82.8)
Sex, male/female, *n*	13/24
Baseline (prior to each course) FEV_1_ predicted, % (range)	76 (42–105)
Age 6–11 years	87 (47–105)
Age 12–17 years	72 (42–97)
Baseline (prior to each course) BMI, kg/m^2^ (range)	18.55 (14.55–25.38)
Age 6–11 years	16.61 (14.55–25.38)
Age 12–17 years	19.41 (16.06–23.66)
Dose, mg per day (range)	300 (100–600)
Age 6–11 years	300 (100–300)
Age 12–17 years	300 (200–600)
Dose, mg/kg per day (range)	6.48 (3.45–14.05)
Age 6–11 years	8.33 (3.45–12)
Age 12–17 years	6.06 (4.11–14.05)
Concentration, mg/L (range)	2.41 (0.2–8.91)
Age 6–11 years	3.08 (0.2–8.91)
Age 12–17 years	2.07 (0.2–4.96)
Sample time after dose, h (range)	1–31
Samples when patient taking PPI or H_2_-receptor antagonist, %	76
Samples when patient taking clarithromycin, %	2
Samples when patient taking rifampicin, %	2
Samples when patient taking rifabutin, %	11
Samples when patient taking Orkambi^®^, %	3
Samples where liver function tests normal, %	40

ABPA, allergic bronchopulmonary aspergillosis; *Aspergillus, A. fumigatus*; *Scedosporium, S. apiospermum* and *L. prolificans*; *Exophiala, E. dermatitidis*; H_2_-receptor antagonist, histamine H_2_-receptor antagonist; PPI, proton pump inhibitor.

### Population pharmacokinetics

**Figure 1.**
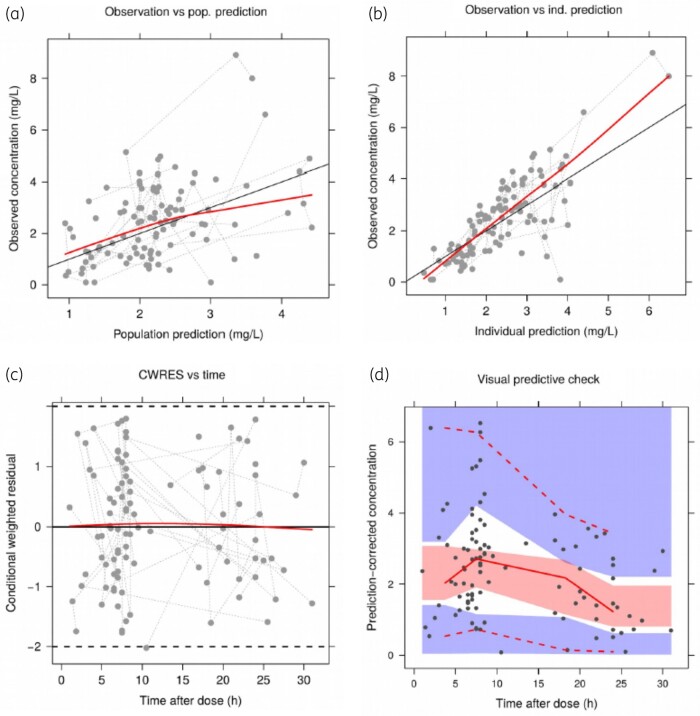
Goodness-of-fit plots for the final model. (a) Population (pop.) predictions versus observations; (b) individual (ind.) predictions versus observations; (c) conditional weighted residuals (CWRES) versus time after dose (time); and (d) visual predictive check showing model-simulated 95% CIs for the simulated 2.5th, 50th and 97.5th percentiles (shaded areas) compared with the observed percentiles (lines). This figure appears in colour in the online version of *JAC* and in black and white in the print version of *JAC*.

The pharmacokinetics of posaconazole in children with CF were adequately described by a one-compartment model with IIV on clearance and additive and proportional residual error. Goodness-of-fit plots, and a visual predictive check indicating an acceptable model fit, are given in Figure 1. Parameter estimates and bootstrap results from the final model are included in Table 2. Of the 1000 bootstrap samples, 5 were omitted due to minimization terminating and 107 with final estimate zero gradients. No covariates were selected during the stepwise covariate modelling. The absorption rate constant (K_a_) was 0.16 h^−1^, apparent clearance (CL/F) was 8.43 L/h and apparent volume (*V*/F) was 186 L, standardized to a 70 kg individual. The corresponding estimates for the reduced dataset (including only those samples with known dose times) were: 0.13 h^−1^, 7.93 L/h and 108 L for K_a_, CL/F and *V*/F, respectively, and hence there was no apparent influence of assuming dose time so all samples were included.

**Table 2.** Parameter estimates from the final model

Parameter	Estimate (%RSE)	IIV %CV (%RSE)	Bootstrap median (95% CI)	Bootstrap IIV %CV (95% CI)
CL/F (L/h)^a^	8.43 (8.7)	38 (36.8)	8.38 (7.11–10.07)	37 (20–52)
*V*/F (L)^a^	186 (55.4)	—	197 (107.96–413.55)	—
K_a_ (h^−1^)	0.16 (55.5)	—	0.15 (0.05–2.09)	—
Proportional error (%CV)	36 (23.2)	—	35 (24–44)	—
Additive error (mg/L)	0.15 (48.4)	—	0.15 (0.02–0.42)	—

%CV, coefficient of variation; RSE, relative standard error.

The NONMEM model code is given in the Supplementary data available at *JAC* Online.

a CL/F and *V*/F allometrically scaled.

### Dose simulations

**Figure 2.**
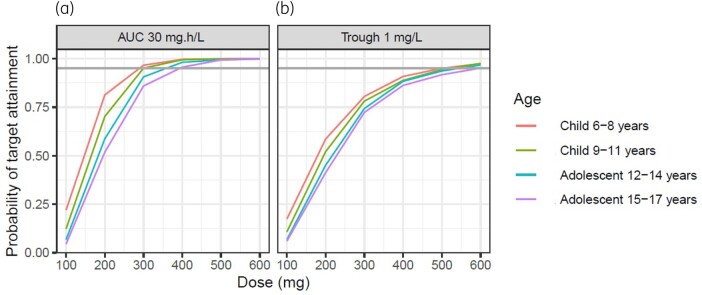
Simulated probability of AUC being 30 mg·h/L (a) or trough concentration being 1 mg/L (b) for OD dosing split by age group. The grey horizontal line represents a 90% PTA. This figure appears in colour in the online version of *JAC* and in black and white in the print version of *JAC*.

**Figure 3.**
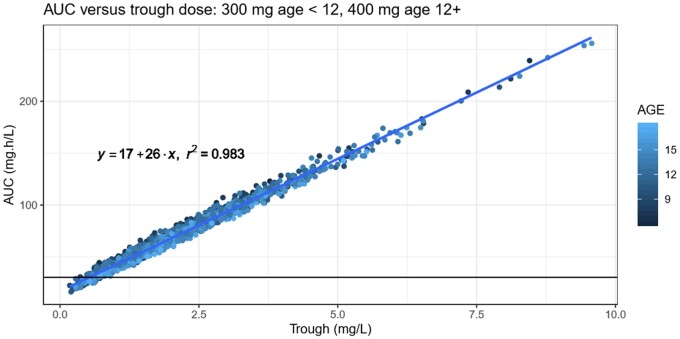
Simulation of AUC versus trough concentration using a dose of 300 mg every 12 h for two doses then 300 mg OD in children <12 years old and 400 mg every 12 h for two doses then 400 mg OD in children aged 12 years and above. Black horizontal line represents an AUC of 30 mg·h/L. This figure appears in colour in the online version of *JAC* and in black and white in the print version of *JAC*.

The simulations using the derived pharmacokinetic parameters from the model demonstrated that by using the tablet formulation the probability of attaining a trough target concentration of 1 mg/L with the standard adult starting dose of 300 mg every 12 h for two doses (loading) followed by 300 mg once daily (OD) was 77% in children aged 9–11 years and 83% in children aged 6–8 years; using a starting dose of 400 mg every 12 h for two doses followed by 400 mg OD, the probability of attaining a trough target of 1 mg/L was 88% in adolescents aged 12–14 years and 86% in adolescents aged 15–17 years. However, simulations using an alternative AUC target showed that with these dosing regimens there was a greater probability (more than 90% in both age groups) of attaining an AUC of 30 mg·h/L. Plots of simulated target attainment versus dose are shown in Figure 2. AUC and trough concentration were highly correlated (r^2^ = 0.98) (see Figure 3) and simulations showed that trough concentrations of >0.75 mg/L would exceed an AUC of 30 mg·h/L in 90% of patients.

## Discussion

Here we report the first population pharmacokinetic analysis of posaconazole gastroresistant tablets in 37 children with CF, 14 of whom were less than 12 years old, who received posaconazole to treat CF-related fungal lung disease. A one-compartment model adequately described the data (Figure 1) and no significant covariates were found. We have made simulations from the model that have suggested an initial starting dose of 300 mg every 12 h for two doses then 300 mg OD in children aged 6–11 years and 400 mg every 12 h for two doses then 400 mg OD in children aged 12–17 years.

Posaconazole has been prospectively studied in 14 children with CF. The study^8^ reported that posaconazole was well tolerated and improvements in lung function were observed. Posaconazole plasma trough concentrations of >1 mg/L were achieved in 100% of children taking the tablet formulation, all of whom were aged 12 years and older and receiving a dose of 300 mg OD. A case study of a 13-year-old male patient with CF treated with posaconazole tablets for *A. fumigatus* infection also demonstrated that plasma trough concentrations of >1 mg/L were attained with a dose of 300 mg, along with improved lung function and eradication of the organism.^24^ However, despite these positive experiences thus far, there have been no published pharmacokinetic data to support dosing specific to children with CF, including younger children, and especially in the context of altered pharmacokinetics in this population.

The population pharmacokinetics of posaconazole in children with CF from our model are broadly in line with the parameters ascertained by Boonsathorn *et al.*^15^ in children without CF who estimated CL/F to be 14.95 L/h and *V*/F to be 201.7 L. Although the CIs of the CL/F value derived from our model (7.11–10.07) are within the stated 95% CIs for CL/F derived by Boonsathorn (6.3–34.1), the lower value in our model may be explained by the difference in formulations used by the patients in each of the models, with lower bioavailability of the suspension used in the model in children without CF leading to a higher estimate of CL/F.^25^ However, our value is similar to the CL/F of the tablet formulation (7.3 L/h) derived from the population pharmacokinetic model described by Petitcollin *et al.*^26^ in adult haematological patients. We had limited data in the absorption phase and our absorption rate constant was somewhat lower than that reported by others (0.16 versus 0.588 h^−1^).^26^ Fixing K_a_ to 0.588 h^−1^ had limited impact on model fit and other parameter estimates (ΔOFV +2.64 points when fixing K_a_), indicating inferences on AUC or trough concentration would not be affected.

Posaconazole is metabolized via UDP glucuronidation (UDP-glucuronosyltransferase, UGT) and is a substrate for p-glycoprotein (P-gp) efflux.^27^ Children with CF often require antibiotics to treat pulmonary infections caused by *Staphylococcus aureus* and non-tuberculous mycobacteria including *Mycobacterium avium* complex. Recommended treatment for these includes rifampicin and rifabutin (inducers of these pathways) and clarithromycin (an inhibitor), which respectively have the potential to either reduce or increase serum concentrations of posaconazole if given concomitantly. Many patients with CF are now treated with CFTR modulators, which target the basic CFTR channel defect. One of these, Orkambi^®^, a combination of ivacaftor and lumacaftor, can reduce posaconazole serum concentrations by induction of UGT via the lumacaftor component, and concomitant use with posaconazole is not recommended.^27^ Posaconazole can also increase exposure of the ivacaftor component of Orkambi^®^, though due to the induction effect of the lumacaftor component of Orkambi^®^ on CYP3A enzymes at steady state the overall exposure of ivacaftor with concomitant posaconazole does not exceed that achieved in patients receiving ivacaftor monotherapy.^28^ Therefore it is not necessary to adjust the dose of Orkambi^®^ if initiating posaconazole in patients already commenced and stabilized on this medicine, as was the case in our cohort. We tested these interacting drugs as covariates in the analysis as it was hypothesized that these may be associated with changes in posaconazole CL/F. However, none were statistically significant. It is likely that the small percentage of samples included within the analysis was insufficient to detect any effects. We also tested the effect of concurrent use of proton pump inhibitors and histamine H_2_-receptor antagonists as a covariate, and no effect on the CL/F of the tablet formulation was seen, confirming the findings already seen in healthy subjects.^29^ Similarly, liver function abnormalities were not found to have an effect on CL/F when tested as a covariate within the analysis. This concurs with findings by Petitcollin *et al.*^26^ who suggested that although baseline ALT levels were negatively correlated with posaconazole clearance, the effect was small, with a 5-fold increase in ALT levels resulting in only a 25% reduction in clearance.

Dosing simulations were carried out against a target trough concentration of 1 mg/L, as per established guidelines,^7^^,^^10^ and an alternative target of an AUC of 30 mg·h/L. The latter target was chosen from the EUCAST Rationale Document for Clinical Breakpoints for *Aspergillus*, which states this value as it is associated with a 75% response rate in patients receiving posaconazole as salvage therapy for invasive aspergillosis and is equivalent to a *C*_avg_ of 1.25 mg/L.^22^^,^^30^ Although neither of these targets is well validated in larger cohorts including those with CF, Gastine *et al.*^23^ confirmed that in a rabbit model of *A. fumigatus* invasive pulmonary aspergillosis, an AUC greater than 30 mg·h/L was associated with adequate resolution of the galactomannan index—a surrogate marker of antifungal response against *Aspergillus* species. For azole antifungals, the pharmacodynamics and thus response to treatment are ultimately related to the MIC of an organism, and specifically the AUC_0–24_/MIC ratio, which is the pharmacodynamic index for posaconazole consistently shown to be most closely correlated with efficacy. Gastine *et al.*^23^ utilized an *A. fumigatus* strain with an MIC of 0.25 mg/L in line with the EUCAST epidemiological cut-off value for this organism, resulting in an AUC_0–24_/MIC ratio of 120. Recommendations for the AUC_0–24_/MIC target range between 100 and 200 for treatment of *A.fumigatus.*^22^^,^^27^^,^^31^

Our simulations showed that an AUC of 30 mg·h/L was easier to attain with ‘standard’ dosing, that is doses in a similar range to those licensed in adults, than a trough concentration of 1 mg/L. With the number of samples required to determine AUC accurately being impractical in clinical use, our simulations showed that with AUC and trough concentrations highly correlated, trough concentrations of >0.75 mg/L (lower than the recommended 1 mg/L target trough concentration for treatment of susceptible fungal diseases) would exceed an AUC of 30 mg·h/L in 90% of patients. Future work is required to evaluate whether a trough concentration of 0.75 mg/L, as a surrogate for AUC, is sufficient to optimize outcome in this cohort for the treatment of *Aspergillus*-related lung disease, and to evaluate response for less susceptible strains of *A. fumigatus* where we would expect to increase the AUC attained to maintain an AUC_0–24_/MIC ratio of >100. Our study also included children with lung disease associated with *Scedosporium* species (*Lomentospora prolificans* and *Scedosporium apiospermum*) and *E. dermatitidis*. Unlike *Aspergillus*, pharmacodynamic targets have not been elucidated for these organisms and there is a need to identify these and produce recommendations on the optimal use of posaconazole in children with CF with these organisms.

This study is limited by its retrospective nature and ideally a prospective study with optimal sample timing should be carried out. However, given the difficulty in recruiting to studies requiring multiple blood samples in children, it is likely that our study included a larger cohort than might otherwise have been possible. Our simulations used recommended, though unvalidated, targets in our cohort of patients. There is a need to refine and validate these according to the organism, site of action and disease state, and most importantly assess the clinical outcome in children with CF.

### Conclusions

Our study confirms that the population pharmacokinetics of posaconazole in children with CF are in line with data published in other paediatric populations. Our dose simulations suggest that a starting dose of 300 mg every 12 h for two doses (loading) then 300 mg OD in children aged 6–11 years and 400 mg every 12 h for two doses then 400 mg OD in adolescents aged 12–17 years will ensure a 90% probability of attaining an AUC of 30 mg·h/L, a target which is known in other patient populations to be associated with a favourable outcome when treating *Aspergillus* infections.^22^^,^^30^ If aiming for trough concentration targets of >1 mg/L, 12%–23% of patients are predicted to require a higher dose.

There is a need to establish pharmacodynamic targets for other fungi commonly found in children with CF and to evaluate clinical outcomes using both these targets, and those already recommended for *A. fumigatus*, including whether a trough concentration of 0.75 mg/L is sufficient to optimize outcome for the treatment of *Aspergillus*-related lung disease.

## Funding

This study was supported by the National Institute of Health Research (NIHR) through a Health Education England/NIHR Pre-doctoral Clinical Academic Fellowship (S.B., NIHR300407), a Senior Investigator Award (J.C.D., NIHR201402) and the Imperial Biomedical Research Centre. J.F.S. and S.G. were supported at an institution level by the NIHR Great Ormond Street Biomedical Research Centre and J.F.S. received a UK Medical Research Council fellowship (MR/M008665/1).

## Transparency declarations

None to declare.

## Supplementary data

The NONMEM model code is available as Supplementary data at *JAC* Online.

## Supplementary Material

dkab312_Supplementary_Data
